# Partial Least Squares, Experimental Design, and Near-Infrared Spectrophotometry for the Remote Quantification of Nitric Acid Concentration and Temperature

**DOI:** 10.3390/molecules28073224

**Published:** 2023-04-04

**Authors:** Luke R. Sadergaski, Sawyer B. Irvine, Hunter B. Andrews

**Affiliations:** 1 Radioisotope Science and Technology Division, Oak Ridge National Laboratory, Oak Ridge, TN 37831, USA; 2 Isotope Processing and Manufacturing Division, Oak Ridge National Laboratory, Oak Ridge, TN 37831, USA

**Keywords:** multivariate analysis, regression, water band, D-optimal design, prediction performance

## Abstract

Near-infrared spectrophotometry and partial least squares regression (PLSR) were evaluated to create a pleasantly simple yet effective approach for measuring HNO_3_ concentration with varying temperature levels. A training set, which covered HNO_3_ concentrations (0.1–8 M) and temperature (10–40 °C), was selected using a D-optimal design to minimize the number of samples required in the calibration set for PLSR analysis. The top D-optimal-selected PLSR models had root mean squared error of prediction values of 1.4% for HNO_3_ and 4.0% for temperature. The PLSR models built from spectra collected on static samples were validated against flow tests including HNO_3_ concentration and temperature gradients to test abnormal conditions (e.g., bubbles) and the model performance between sample points in the factor space. Based on cross-validation and prediction modeling statistics, the designed near-infrared absorption approach can provide remote, quantitative analysis of HNO_3_ concentration and temperature for production-oriented applications in facilities where laser safety challenges would inhibit the implementation of other optical techniques (e.g., Raman spectroscopy) and in which space, time, and/or resources are constrained. The experimental design approach effectively minimized the number of samples in the training set and maintained or improved PLSR model performance, which makes the described chemometric approach more amenable to nuclear field applications.

## 1. Introduction

Near-infrared (NIR) spectrophotometry has been used for the rapid, nondestructive analysis of numerous species in many food and pharmaceutical industrial applications [1,2]. Implementing optical techniques (e.g., NIR) for in situ measurements to support production operations generally improves processing speed, efficiency, and reproducibility. Online monitoring provides the benefit of real-time feedback to optimize system performance and help guide process decisions during chemical operations [3]. There are few examples of such technology fully implemented in the nuclear field at the industrial scale because of the complexity associated with radiological constraints [4,5,6,7]. The nuclear fuel cycle and radioscope production processes would greatly benefit from the implementation of online monitoring to minimize waste, enhance worker safety, and track material inventory in real time [8].

Fully integrated spectroscopic monitoring examples in the nuclear field are sparse in part owing to the harsh and restrictive environments often needed to deploy such technologies (e.g., hot cells) [6,7,9]. Two important variables, including (1) temperature and (2) resources (i.e., number of samples), are often overlooked in many laboratory-scale proof of principle studies [10,11,12]. These variables must be accounted for when such technologies are implemented in harsh and restrictive environments such as radiochemical hot cells or caves. Although other popular examples of optical spectroscopy (e.g., Raman) can be used for the quantification of HNO_3_ concentration and temperature, these techniques often require additional safety protocols, such as laser shields [13,14]. NIR spectrophotometry, which uses an incoherent light bulb for analysis, is readily deployable in remote settings, is self-referencing, and does not require additional safety measures in an environment that is already inundated with numerous regulations.

NIR water absorption bands (750–2500 nm) have been studied for the purposes of fundamental and applied research [15,16,17,18,19]. Water structure is highly sensitive to changes in temperature and solute interactions. For example, strong acids (e.g., HNO_3_) dissociate into protons (H^+^) and corresponding anions (i.e., NO_3_^−^), which perturb the local H-bonding network, giving rise to spectral variations in NIR water bands [20,21,22,23,24,25]. Thus, aqueous species that do not absorb NIR radiation themselves but interact with water molecules can be quantified. Additionally, water structure is so sensitive to temperature fluctuations that this variable alone renders the quantification of solute species challenging.

Covarying NIR spectral features cannot be quantified using univariate approaches such as Beer’s law [25]. Multivariate analysis, or chemometrics, can correlate covarying NIR spectral signatures to analyte concentration. One popular example, partial least squares regression (PLSR), is a statistical approach used to relate the independent (*X* matrix) and dependent (Y matrix) variables with linear combinations of latent variables (LVs) in multicomponent systems. PLSR is a supervised regression technique that depends on a training set that includes all spectrally active species covering the breadth of anticipated conditions. The training set comprises calibration and validation samples. The calibration samples are used to build the regression model, and the validation set contains samples not included in the model-building phase to assess prediction performance. These samples are often selected using a subjective one-factor-at-a-time approach, which normally results in numerous samples not amenable to many hot cell applications [9,26]. Recent work has established D-optimal designs as a statistical framework for selecting representative training set sample concentrations while minimizing the number of samples without weakening PLSR model prediction performance or increasing bias [25,27,28,29]. This approach may benefit optical measurements taking place in harsh, restrictive, and expensive environments (e.g., a hot cell). To the best of the authors’ knowledge, optimal designs have not been used to simultaneously choose concentration and temperature levels within a given factor space until now.

This research evaluates D-optimal sample design, NIR spectrophotometry, and PLSR for the quantification of HNO_3_ concentrations (0.1–8 M) and temperatures (10–40 °C). These conditions are highly applicable to validating sample compositions and monitoring process streams for the Ac-225 Program at Oak Ridge National Laboratory, as well as applications in the nuclear field and other industries. Three points of scientific advancement are covered in this work: (1) multivariate analysis enables quantitative HNO_3_ concentration and temperature predictions based solely on NIR spectra, (2) a D-optimal design with a cubic order model was used to minimize temperature and concentration levels in the training set, and (3) PLSR prediction performance was verified with flow tests and the Hotelling’s T^2^ statistic identified outlier samples from unanticipated spectral artifacts. The D-optimal design can effectively minimize resource (i.e., time and material) consumption and generate a PLSR model suitable for the intended use. The new modeling approach can quantitatively measure acid concentration and temperature in a remote setting without any prior knowledge or without destroying the sample. This work overcomes drawbacks associated with the application of chemometric methods to help pave the way for optical spectroscopy applications in the nuclear field.

## 2. Results and Discussion

### 2.1. Absorption Spectra

Intense water absorption bands occur near 1450 and 1940 nm in the NIR region of the electromagnetic spectrum. The water band centered near 1450 nm is due to the combination of symmetric and antisymmetric O–H stretching modes (i.e., first overtone). The dynamic behavior of this band has been studied in detail. Additional NIR regions (e.g., 1100–1300 nm and 1800–2100 nm) have also been used to study water structure and develop regression models for quantitative analysis [9,15,16]. The intensity for the first overtone band requires a spectrophotometer with a high dynamic range or a smaller optical path length cell for quantification (e.g., 1 mm). Blanking in the air is advantageous because this reference is independent of the sample temperature. However, great caution should be taken to avoid bubbles in the cuvette or drying out the sample to the point of precipitation. This approach will be compared to referencing the spectrophotometer to solutions at various temperatures in future work.

**Figure 1:**
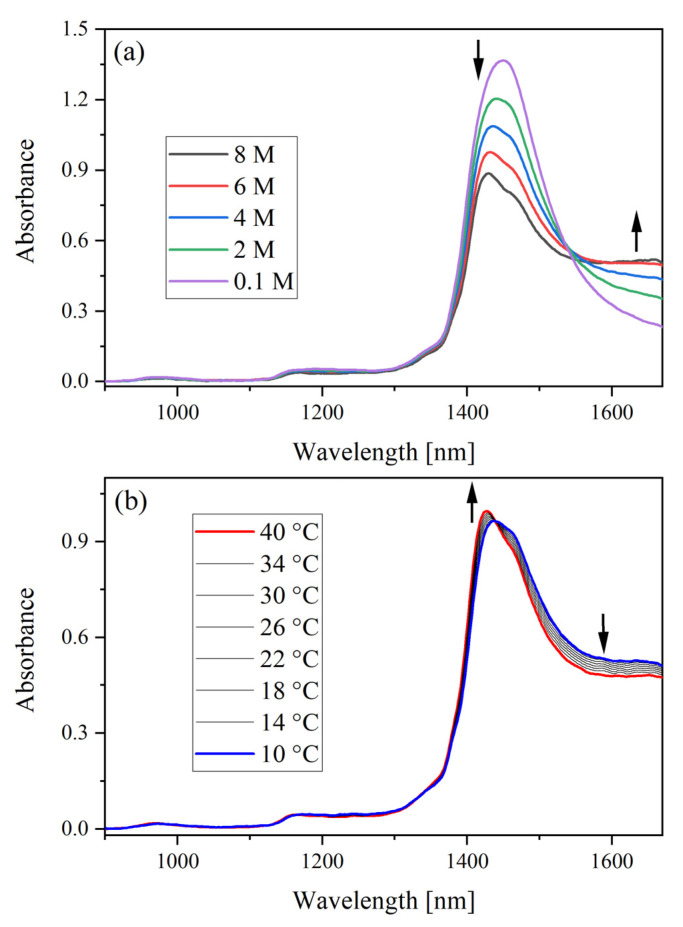
NIR absorbance spectrum of (**a**) 0.1–8 M HNO_3_ at 24 °C and (**b**) 6 M HNO_3_ from 10–40 °C. Arrows note the direction of spectral change as the concentration or temperature was increased. The spectrometer was blanked in air, and the optical path length was 1 mm.

NIR spectra (900–1670 nm) with HNO_3_ concentrations (0.1–8 M) and temperatures (10–40 °C) are shown in Figure 1. The absorption bands near 970 and 1190 nm had weak signal intensity with the 1 mm cuvette. These bands could be used for quantification with a longer optical pathlength cuvette [23]. The band centered near 1440 nm, assigned to the combination of symmetric and antisymmetric O–H stretching modes (first overtone), dominated the spectrum [14,15]. With increasing HNO_3_ concentration, the net absorbance near 1400 nm decreased, and the absorbance above 1540 nm increased. An apparent isosbestic point was identified (Figure 1a) near 1540 nm until the concentration was greater than 6 M HNO_3_. This is due to the concentration-dependent equilibrium between bonded and nonbonded O–H valences [24]. The H_3_O^+^ and NO_3_^−^ species present in the system owing to the dissociation of HNO_3_ are order-producing and order-destroying, respectively. Another isosbestic point caused by temperature was identified near 1440 nm, which is consistent with previous reports. This point is related to the weakening of intermolecular H-bonds, which decrease absorption greater than 1440 nm with increasing temperature and the strengthening of covalent O–H bonds, which increase absorption below 1440 nm. This results in an overall blue shift to shorter wavelengths (i.e., higher energy). This interpretation was derived from a two-state mixture model in which one component converts to another as a function of temperature [15].

Each ion in an aqueous solution has a unique fingerprint on the NIR water band(s). Distinct differences in spectral variations exist even between cations of the same charge (e.g., Na^+^ and K^+^) [16]. Limits of detection for relevant species such as Na^+^, which could be encountered in production operations, are near 42 mM (~1000 mg mL^−1^) [24]. Although many species may be present in these solutions, a large number may be negligible. Future studies may need to include additional components in the regression model (e.g., Na^+^, Fe^3+^), which further motivates the reason why the designed approach for minimizing the number of training set samples is crucial.

### 2.2. D-Optimal Design

D-optimal design was chosen to select continuous analyte concentrations and temperature levels (Table 1) for the multivariate regression model training set. The acid and temperature levels were expected to cover the anticipated conditions. A higher-order model (i.e., cubic) was used to approximate the true response surface of this training set, which included temperature. With a larger number of factors, higher-order models may also be necessary. Although quadratic models are commonly used to estimate analyte concentrations, this study hypothesized that a higher-order model would be necessary to account for temperature fluctuations.

The designed approach in this study was useful for minimizing the number of samples in the training set and selecting samples within a statistical framework void of user bias. This approach may also be advantageous over calibration transfer functions when the conditions between laboratory and in-field measurements are significantly different [30]. In total, 15 samples were included in the calibration set to test how few samples could be used to build the PLSR model. The additional five lack-of-fit points were used in the validation set to test model performance with additional temperature levels. This number was chosen based on evaluating the fraction of design space (0.98), which indicates satisfactory coverage of the factor space [14,31]. Spectra were also collected at five temperature levels for each concentration at nearly even intervals (e.g., 10 °C, 18 °C, 24 °C, 32 °C, and 40 °C), and samples 3 (6.025 M HNO_3_) and 13 (2.075 M HNO_3_) were measured at 2 °C intervals. Temperature levels could be included in future D-optimal designs as discrete intervals, which is an option in the software, if more points are required.

### 2.3. Partial Least Squares Regression

PLSR was used to find correlations between analyte concentrations and temperatures by modeling the spectral features shown in Figure 1. Preprocessing and feature selection can greatly improve the regression analysis. This study applied a preprocessing strategy to optimize the regression analysis. The uncertainty in PLSR models that results in a systematic or random error in model parameters is based primarily on variance and bias. Variance contributes the most to uncertainty in a model that is too complex (i.e., comprised of too many samples). Alternatively, bias tends to dominate the uncertainty in a model if too few samples are included. The number of samples in the calibration has a substantial effect on model performance. The D-optimal calibration set contained 15 samples (Table 1), the extended calibration set (ECal) contained 33 samples, and the validation set contained 50 samples. PLSR models were built using the D-optimal set and ECal and were used to predict the concentrations and temperatures of samples in the validation set (i.e., samples not included in the training set).

Standard PLS2 models were used to calibrate the system for HNO_3_ concentration and temperature. These models were preprocessed with only a simple baseline offset and an SG smoothing step to remove instrument noise accumulated over time. The spectral regions were trimmed after recalculating the model with only the prominent regression coefficients identified by modeling the entire spectrum. PLS2 model regression coefficients can be found in the Supplementary Materials (Figure S1). This region (1240–1700 nm) consisted of the entire water band centered near 1440 nm. Regression coefficients summarized the relationship between the predictors (wavelengths) and the response (concentration). Variables with large regression coefficients—positive or negative—played an important role in the model by affecting the response variables in the prediction.

To optimize the regression, PLS1 models were generated for acid concentration and temperature independently using the D-optimal set and ECal with additional preprocessing strategies and a genetic algorithm for feature selection [29]. The preprocessing step for the acid determination consisted of an SG smoothing algorithm with a seventh-order polynomial and 61 smoothing points (i.e., 30 left/right). The temperature PLS1 models were built with spectra that were processed by SNV to remove scattering and an SG second derivative with a third-order polynomial and 41 smoothing points (i.e., 20 left/right). The features selected by the GA and the explained variance and *RMSE* for each generation are shown in Figures S2 and S3.

A summary of calibration, cross-validation, and prediction statistics is shown in Table 2. The preprocessing and feature selection PLS1 D-optimal (D-opt. in the table) models had slightly lower *RMSEP* and *RMSEP*% values for each variable compared to the ECal models and PLS2 models. However, the improvements may not outweigh the additional complexity for end users running two models simultaneously. Minimal improvements in *RMSEP*% confirm that there are likely no “goldilocks” preprocessing/feature selection options, which suggests that the spectra are relatively simple and can be modeled near the true optimum without much trial and error [29]. The D-optimal PLS2 and PLS1 models for HNO_3_ lowered the most; the *RMSEP*% decreased by 33% from 2.1 to 1.4%. In general, the *RMSEP* and *RMSEP*% values for the models built using the D-optimal PLS1 and PLS2 models were lower than the ECal, which contained many more samples. The D-optimal model bias values were generally similar to or closer to zero than the ECal models. This result suggests that the designed approach, which contained only 15 samples, can capture the structured variation in the data set without increasing bias.

The *RMSEC*, *RMSECV*, and *RMSEP* statistics for the ECal models were more balanced than the D-optimal models. This balance suggests that the D-optimal approach successfully minimized the samples in the training set because during full cross-validation, leaving samples out significantly decreased the prediction capability. Therefore, fewer than 15 calibration samples are unlikely to adequately model this factor space. The D-optimal *RMSEC* and *RMSEP* values were consistent, which suggests that the model can describe new data well. Cross-validation statistics for the D-optimal set may not provide an accurate indication of model performance [24].

**Figure 2:**
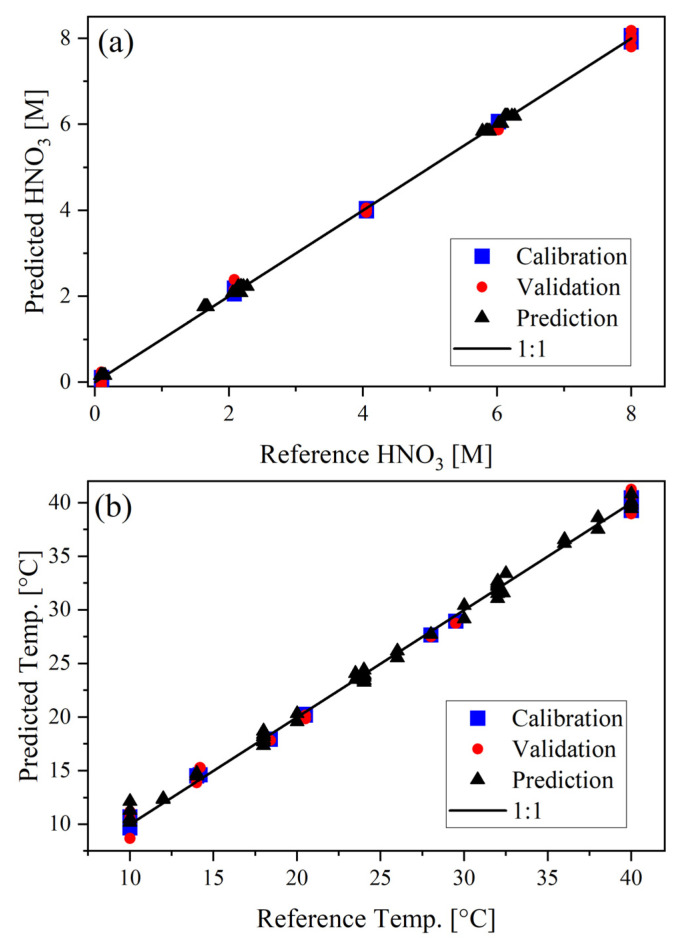
Calibration, cross-validation, and prediction parity plots for HNO_3_ (**a**) concentration (M) and (**b**) temperature (°C). The model was built using the D-optimal calibration set and 4 LVs.

The PLS1 models built using the D-optimal calibration samples had the lowest *RMSEP*% for HNO_3_ concentration (1.4%) and temperature (4.0%). A parity plot for the calibration, cross-validation, and prediction performance is shown in Figure 2. The predicted values fall near the 1:1 line, which suggests good model performance. For each model presented in Table 2, more than 99% of the Y-variance was explained and both R^2^ and Q^2^ values were greater than 0.99 (Table S1).

### 2.4. Real-Time Tests

**Figure 3:**
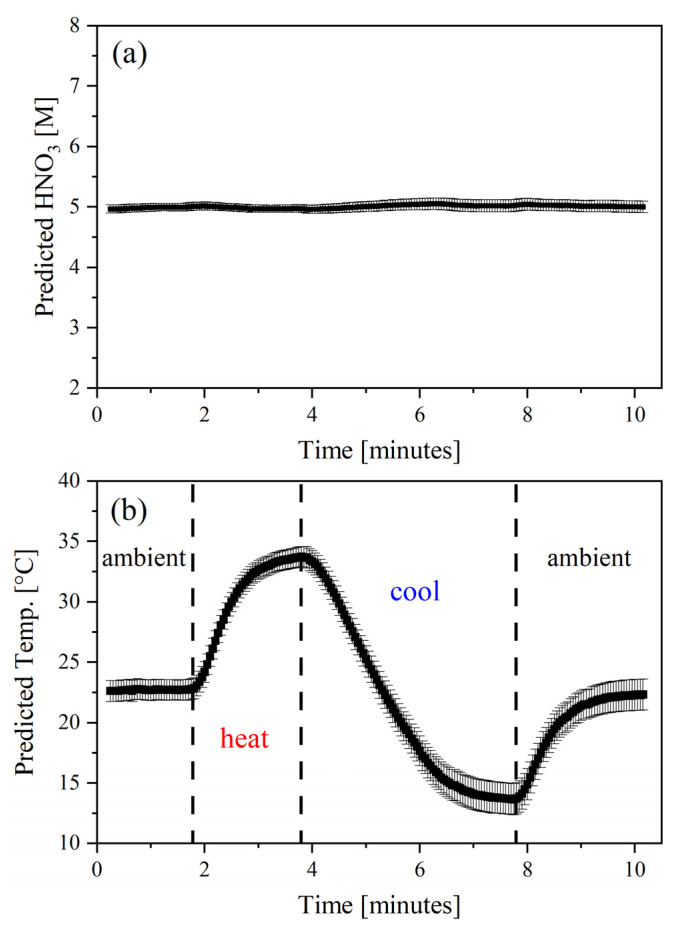
PLSR predicted HNO_3_ (**a**) concentration (5 M) and (**b**) temperature (Temp., °C) during flow experiments.

The PLS2 model was used to predict the HNO_3_ concentration and temperature of flow test samples. The first test simulated varying temperature profiles when acid concentration was held constant to ensure that HNO_3_ predictions were not dependent on temperature fluctuations (Figure 3). Spectra were collected at 1 s intervals, but the average of three (i.e., 3 s intervals) is shown. In this test, 5 M HNO_3_ was pumped through the syringe at a rate of approximately 1 mL min^−1^ while spectra were collected at 1 s intervals and ambient temperature (~22.5 °C). Then, the temperature-controlled cuvette holder was set to 40 °C. This holder heated the sample to nearly 34 °C for approximately 2 min. The temperature did not stabilize because the room-temperature HNO_3_ solution was continuously being pumped through the system. Then, the temperature-controlled cuvette holder was set to 10 °C. The sample in the cuvette holder cooled to nearly 13 °C but did not reach a steady state after approximately 5 min. Finally, the sample was brought back to room temperature (~22.5 °C). The percent relative standard deviation of HNO_3_ predictions was 0.5%, which indicates exemplary model performance despite fluctuating temperatures. The average reported deviation of approximately 0.081 M was consistent with the *RMSEP* of 0.082 M reported in Table 2. *RMSEP* generally provides an estimate of the deviation in the predicted sample concentrations. This flow test suggests that the temperature deviations anticipated during process operations will not disrupt HNO_3_ predictions. The small and consistent deviation in the predicted HNO_3_ concentration and temperature values also indicated that the model could handle incremental temperature gradients between points elected by the D-optimal design in the calibration set.

The second flow experiment tested the model’s ability to predict HNO_3_ concentrations between the points in the factor space included in the PLSR model and how well the model could handle outliers created by bubbles in the line. Air was intentionally introduced in the line to create samples representative of off-normal conditions and test model boundaries. Spectra were collected at 2 s intervals during this experiment. The test began with flowing 8 M HNO_3_ through the flow cuvette for approximately 2 min. Partway during this exercise, the tubing was lifted out of the feed solution for approximately 15 s to allow air to enter the line. Then, the tubing was placed back in the 8 M HNO_3_. DI water was added to the 8 M HNO_3_ solution with a second pump operating at 2.5 mL min^−1^ while it was mixed on a stir plate, and the first pump introduced the mixture into the flow cuvette at a rate of approximately 0.8 mL min^−1^. This addition of DI water created an HNO_3_ concentration gradient from 8 M to approximately 0.4 M after 15 min of operation. The tubing was also intentionally removed from the mixture for 15 s twice as the low acid concentration was approached. Then, the sample was switched back to 8 M HNO_3_.

**Figure 4:**
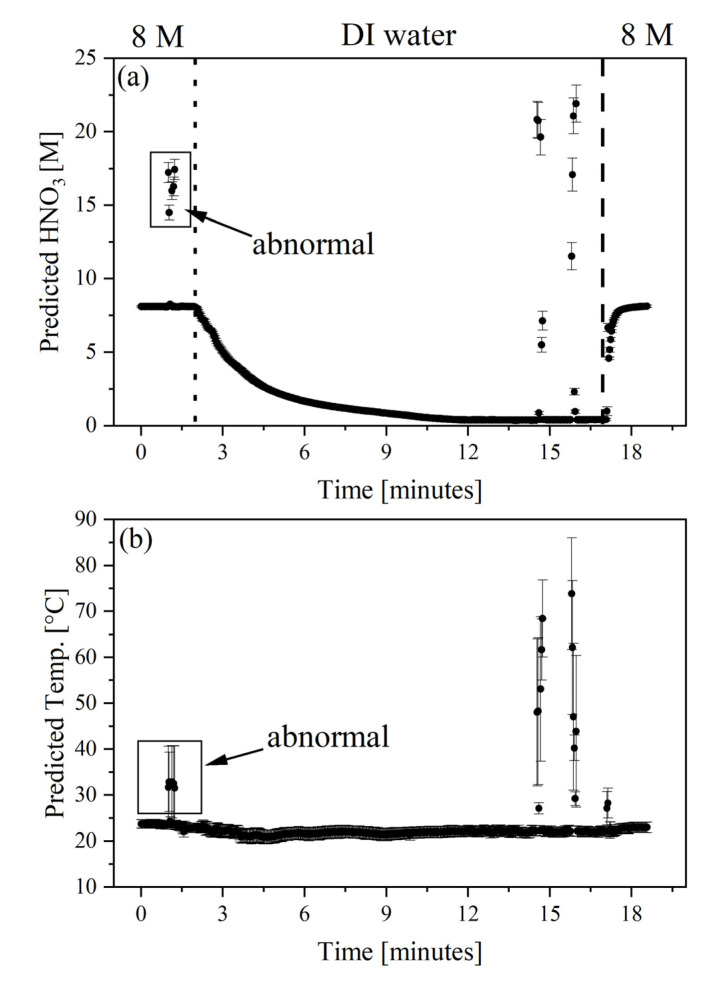
PLSR predicted HNO_3_ (**a**) concentration and (**b**) temperature (Temp., °C) during gradient flow experiments. The vertical dotted line indicates when mixing with DI water was initiated, and the vertical dashed line indicates the switch back to 8 M HNO_3_. Several outliers owing to bubbles are noted.

The average deviation in predicted values was 0.09 M with a percent relative standard deviation of 14%. The measured temperature profile shows encouraging results with an average temperature of 22.2 °C, a standard deviation of 1.2 °C, and a percent relative standard deviation of 13%. These values exclude outliers. Several predicted HNO_3_ concentration and temperature outliers are noted in Figure 4. These sample measurements coincided with the times researchers expected to see bubbles in the line. These predictions fall outside the expected profile concentrations and temperatures but for a justifiable reason. The average deviation associated with the five samples identified in Figure 4a,b were 0.63 M and 8.1 °C. Outliers will be expounded upon in the next section. These results indicate highly precise predictions and show that there are no singularities in the spectral response as a function of acid concentration. Reported deviation and residual variance for each point suggest that the PLSR analysis accurately modeled each point within the factor space.

### 2.5. Outlier Detection

A Hotelling’s T^2^ statistic with a critical limit based on an F-test (*p* value of 5%) was used to identify outliers or situations where the acquisition parameters were operating within or outside of normal conditions. To trust a prediction, it must not be too far from the calibration samples. This statistic compares the variance in each sample to the total variance captured by the LV. Hotelling’s T^2^ distance measures how far the projection of the new samples is from the center of the multivariate space.

**Figure 5:**
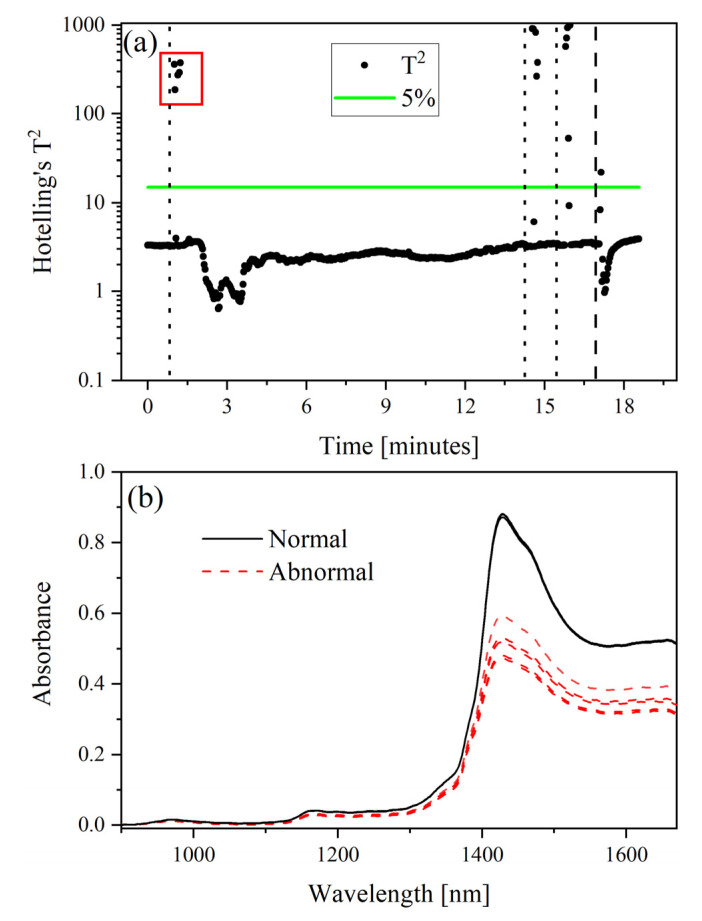
Acid gradient: (**a**) Hotelling’s T^2^ statistic with (green line) *p* value 5% critical limit on a log_10_ scale and (**b**) spectra representing normal and abnormal conditions during the time frame indicated by the red rectangle in (**a**). The vertical dotted lines indicate when air was intentionally introduced into the system, and the vertical dashed line indicates when the sample was switched back to 8 M HNO_3_.

Measured Hotelling’s T^2^ values are shown in Figure 5a. These data points correspond to the samples shown in Figure 4. As expected, most of the measurements fall within the 95% confidence band. This suggests that the PLSR can accurately describe these spectra, which correspond to many acid concentrations between design points of the calibration set. Samples that fall above the 5% critical limit (green line) can be considered outliers. Several outlier spectra are shown in Figure 5b. These samples correspond to spectra of samples with bubbles that were introduced intentionally. Although these resemble the normal spectra, the overall intensity is much lower than the expected spectral signatures. The spectra with bubbles were essentially compressed after the baseline offset correction was applied. Outliers in Figure 5a correlate to samples in Figure 4 that were measured with much larger than average uncertainties.

This figure shows that the model can indicate when samples are outside of normal operating conditions. Events such as bubbles in the line are anticipated in real process samples [6]. Thus, the equipment must be set up so that bubble formation is minimized, and the researcher can identify abnormal conditions. Hotelling’s T^2^ statistic can be used to flag unanticipated conditions (e.g., bubbles) in unknown samples. This criterion should be evaluated in combination with predicted concentrations and sample deviations when guiding process decisions. This approach could also be compared or combined with other optical techniques [32].

## 3. Methods

### 3.1. Sample Preparation

All chemicals were commercially obtained (American Chemical Society–grade) and used as received unless otherwise stated. Concentrated HNO_3_ (70%) and NaNO_3_ were purchased from VWR Life Science. All solutions were prepared using deionized (DI) water with a resistivity of 18.2 MΩ cm at 25 °C. Training set samples contained HNO_3_ (0.1–8 M) to cover the concentration range expected in anion exchange column runs. Samples were prepared gravimetrically by pipetting the appropriate volumes of DI water and HNO_3_ into volumetric glassware.

### 3.2. Absorption Measurements

NIR spectra were collected using an Ocean Insight NIRQuest spectrophotometer with a 100 ms integration time and five-scan average. Triplicate spectra were recorded every 1.65 nm from 897–1711 nm and processed using OceanView software (Ocean Insight, Orlando, FL, USA). The spectrophotometer was referenced to air between each measurement or at the beginning of a series of measurements. Multimode optical fibers with a 400 μm core diameter were used to direct the incoherent light source (360–2600 nm) made by Thorlabs (SLS201L) to the sample and resulting signal to the spectrophotometer. A flow cuvette with a 1 mm optical path length was purchased from Starna Cells Inc. (583.65-Q-1/Z15). A modified Quantum Northwest qpod 2e temperature-controlled sample compartment holder purchased from Avantes was necessary to accommodate the cuvette’s *Z*-height of 15 mm. Two quantum cascade laser–UV collimating lenses were placed on opposite sides of the sample compartment. NIR measurements were performed at varying temperatures (10–40 °C). The cuvette holder has a reported accuracy of ±0.05 °C. Sample solutions were thermally equilibrated in the temperature-controlled environment for approximately 2 min before recording each spectrum. To test the effect of lamp and detector fluctuations on spectral signatures, reference spectra were collected at the beginning of sample acquisitions and between each sample measurement.

A Fluid Metering, Inc. pump with 1/16 in. tubing was used to flow solutions through the cuvette, then paused to collect static reference spectra for model development. For the concentration gradient, two Fluid Metering, Inc. pumps with 1/16 in. tubing bore kits were operated at different flow rates (1 mL min^−1^ and 2.5 mL min^−1^). A beaker with 3 mL 8 M HNO_3_ was pumped into the cuvette at 0.8 mL min^−1^ while the other pump added DI water to the beaker at a rate of 2.5 mL min^−1^. The solution was mixed with a stir bar and stir plate during the measurements. The time was recorded, and volumes of DI water and sample were measured using volumetric glassware.

### 3.3. Experimental Design

The Design of Experiments tool kit in the Unscrambler software package by Camo Analytics (version 11.0.5.0) was used to build a D-optimal design and statistically derive the training set. The D-optimality criterion is designed to estimate the effects of the factors by maximizing the determinant of the information matrix X’X [33]. The design comprised two numeric factors: HNO_3_ (0.1–8 M) and temperature (10–40 °C) and a cubic base order for the design. This resulted in 10 required model points, which were augmented with 10 lack-of-fit (LOF) points. The required model points are the minimum number of samples necessary to estimate the coefficients in the model. A quadratic model is commonly used for selecting analyte concentration levels. A higher-order cubic model was used to estimate concentration and temperature levels. Only six model points are required for a quadratic model to estimate the factor space of two numeric analytes [24]. Optimal response surface designs provide numerous benefits compared with other options (e.g., factorial designs) [31,34]. These options include different high and low values for mixture components, mixture, and process variables in the same design, two independent mixtures in the same design, constraints, and factor limits, various model orders to minimize the number of samples, and combinations of each of these.

### 3.4. Partial Least Squares Regression

PLSR analysis was performed using the Unscrambler X (version 10.4) software package from CAMO Software AS. PLSR models were built from spectra collected on stationary samples. The root mean square error (*RMSE*) of the calibration (*RMSEC*) and cross-validation (*RMSECV*) were used to evaluate calibration statistics. The *RMSECV* residual variance was calculated using a full cross-validation, where each sample was randomly left out of the model. *RMSECV* had the same units as the *Y* variables, and it provided an estimate of the residuals (i.e., uncertainty) in the predictions. The primary validation statistics were *RMSE* of the prediction (*RMSEP*) and percent *RMSEP*. *RMSECV* and *RMSEP* values that are similar indicate a balanced PLSR model. LVs, or factors, were chosen by the last significant improvement in *RMSEC* or *RMSECV*. Adding too many LVs can overfit the model and introduce unwanted noise artifacts.

Proper validation is important to test the dependence of the model on unknown samples and evaluate the predictive power of the regression models. *RMSE*s for the calibration, validation, and prediction were calculated using Equation (1):

(1) $$RMSE=\sqrt{\frac{\sum_{i=1}^{n}{\left({\hat{y}}_{i}-y_{i}\right)}^{2}}{n},}$$

where ${\hat{y}}_{i}$ is the predicted concentration, *y_i_* is the measured concentration, and *n* is the number of samples. The percent *RMSEP* (*RMSEP*%) compares the predicted values with the range. *RMSEP*% was calculated by dividing the *RMSEP* by the median model values using Equation (2):

(2) $$RMSEP\%=\frac{RMSEP}{y_{med}}\times 100\%,$$

where *y_med_* represents the median of each analyte concentration range. *RMSEP*% values ≤ 5% indicate satisfactory model performance.

The deviation (i.e., uncertainty) in *y*-values (i.e., concentrations) predicted by PLSR for each sample was estimated as a function of the global model error, sample leverage, and residual *x*-variance [35]. Hotelling’s T^2^ statistic was used to test the model performance on unexpected conditions with a default *p* value of 5%. This statistic is a powerful indicator of normal or abnormal conditions.

### 3.5. Preprocessing and Feature Selection

A recently developed preprocessing and feature selection strategy was used to optimize model performance [29]. PLSR models were optimized by minimizing the *RMSE*. All spectra were processed with a simple baseline offset correction, which subtracts the lowest point in the spectrum from each variable. Then, several preprocessing transformations were applied to the data set, including scatter (standard normal variate (SNV)), smoothing/derivatives (Savitsky–Golay (SG)), and scaling (mean centering) corrections. Zero, first, or second derivatives were tested with different polynomial orders and left/right smoothing points.

A genetic algorithm (GA) was employed in this work for feature selection [36]. The script was developed in Python and described elsewhere [29]. A GA iteratively forms and tests filters that either block a feature or let it into a model for regression. Based on how well a filter performs, it is either retained, used to make more filters, or discarded before the next generation. Over the course of the generations, the GA should reach an optimal filter. The filters developed for this study used a 6 nm resolution, and the best filter out of five GA runs (150 generations each) was selected as the final result. The inverse of each finalized GA filter was also tested; if the inverse filter resulted in improved or equivalent prediction performance, then the GA simply reduced the dimensionality of the data, permitting better PLSR performance. The optimized transformations and features were used to build PLSR models with the Unscrambler.

## 4. Conclusions

NIR spectrophotometry, design of experiments, and PLSR can be used to model acid concentration and temperature fluctuations with efficiency, high accuracy, and precision. The range of HNO_3_ concentrations (0.1–8 M) and temperature (10–40 °C) are highly applicable to aqueous processing operations in the nuclear field. This work minimized the number of samples required in the training set to save time and resources, which is an essential aspect to consider when implementing such technology in restrictive radiological environments. PLSR predictions of spectra collected during real-time flow demonstrations indicated that the D-optimal design effectively sampled the factor space and that the models built from static spectra and be applied to dynamic samples. Future work will implement this approach for remotely monitoring HNO_3_ concentration in feed adjustments, anion exchange column effluent, and product bottles and measure the temperature of process solutions.

## Figures and Tables

**Table 1:** D-optimal selected HNO_3_ and temperature levels with space and build types.

Run	Acid (M)	Temp. (°C)	Space Type	Build Type
1	1.77	32.5	Interior	Model
2 *	**8.0**	**40.0**	**Vertex**	**Model**
3 *	**6.025**	**14.5**	**Interior**	**Lack of Fit**
4 *	**0.10**	**10.0**	**Vertex**	**Model**
5 *	**0.10**	**20.5**	**Edge**	**Model**
6	5.83	23.5	Interior	Lack of Fit
7 *	**2.075**	**40.0**	**Edge**	**Lack of Fit**
8 *	**8.0**	**28.0**	**Edge**	**Lack of Fit**
9 *	**4.05**	**10.0**	**Center Edge**	**Model**
10 *	**8.0**	**20.5**	**Edge**	**Model**
11	0.15	28.0	Interior	Lack of Fit
12 *	6.025	40.0	Edge	Lack of Fit
13 *	2.075	14.2	Interior	Lack of Fit
14 *	**8.0**	**10.0**	**Vertex**	**Model**
15 *	4.05	29.5	Interior	Lack of Fit
16	2.23	23.5	Interior	Lack of Fit
17 *	4.05	18.4	Interior	Lack of Fit
18 *	**0.10**	**40.0**	**Vertex**	**Model**
**19**	**6.34**	**32.35**	**Interior**	**Model**
20 *	**4.05**	**40.0**	**Center Edge**	**Model**

Required model points are bolded. The asterisk (*) indicates 15 samples included in the calibration set. Temp.: temperature.

**Table 2:** Model calibration and validation statistics.

Model	LVs	*RMSEC*	*RMSECV*	*RMSEP*	*RMSEP*%	Bias
**HNO_3_ D-opt. PLS2**	**4**	**0.053**	**0.086**	**0.083**	**2.10%**	**−0.033**
**Temp. D-opt. PLS2**	**5**	**0.36**	**1.12**	**0.73**	**4.87%**	**0.0013**
HNO_3_ ECal PLS2	4	0.054	0.065	0.082	2.08%	−0.035
Temp. ECal PLS2	5	0.42	0.69	0.87	5.80%	0.40
**HNO_3_ D-opt. GA**	**4**	**0.050**	**0.14**	**0.055**	**1.39%**	**−0.016**
Inverse GA	4	0.044	0.076	0.080	2.03%	−0.032
HNO_3_ ECal GA	4	0.044	0.057	0.068	1.72%	−0.018
Inverse GA	4	0.044	0.057	0.072	1.82%	−0.024
**Temp. D-opt. GA**	**4**	**0.44**	**0.81**	**0.62**	**4.13%**	**0.12**
Inverse GA	4	0.44	0.92	0.85	5.67%	−0.12
Temp. Ecal GA	4	0.41	0.56	0.70	4.67%	0.10
Inverse GA	4	0.44	0.69	0.66	4.40%	0.050

Final models were scaled (i.e., mean centered). Scatter and scaling refer to SNV and mean centering. Bolded text highlights the best PLS2 and PLS1 models.

## Data Availability

Not applicable.

## Supplementary Materials

The following supporting information can be downloaded at: https://www.mdpi.com/article/10.3390/molecules28073224/s1, Figure S1: Regression coefficients for HNO_3_ (a) concentration factor-4 and (b) temperature factor-5. The wavelengths with the most importance are outlined; Figure S2: GA results for the HNO_3_ model. The top two plots show the change in explained variance and *RMSE*, respectively, of the best filter in each generation of the GA. The bottom plot shows the spectral regions selected by GA for acid after preprocessing (smoothing); Figure S3: GA results for the temperature model. The top two plots show the change in explained variance and *RMSE*, respectively, of the best filter in each generation of the GA. The bottom plot shows the spectral regions selected by GA for temperature after preprocessing (scatter correction and smoothing/derivative).
