# Supplementary material for: Partial Least Squares, Experimental Design, and Near-Infrared Spectrophotometry for the Remote Quantification of Nitric Acid Concentration and Temperature
Source: Molecules. 2023 Apr 4;28(7):3224. doi: 10.3390/molecules28073224 (PMC10096128; doi:10.3390/molecules28073224)
Supplement: Supplementary file 1 [file molecules-28-03224-s001.zip › molecules-2289037-supplementary.pdf]

Partial Least Squares, Experimental Design, and Near-Infrared Spectrophotometry for the Remote Quantification of Nitric Acid Concentration and Temperature

Luke R. Sadergaski [1,*], Sawyer B. Irvine [2] and Hunter B. Andrews [1]

[1] Radioisotope Science and Technology Division, Oak Ridge National Laboratory, Oak Ridge, TN 37831, USA
[2] Isotope Processing and Manufacturing Division, Oak Ridge National Laboratory, Oak Ridge, TN 37831, USA
* Correspondence: sadergaskilr@ornl.gov; Tel.: +1-865-574-1167

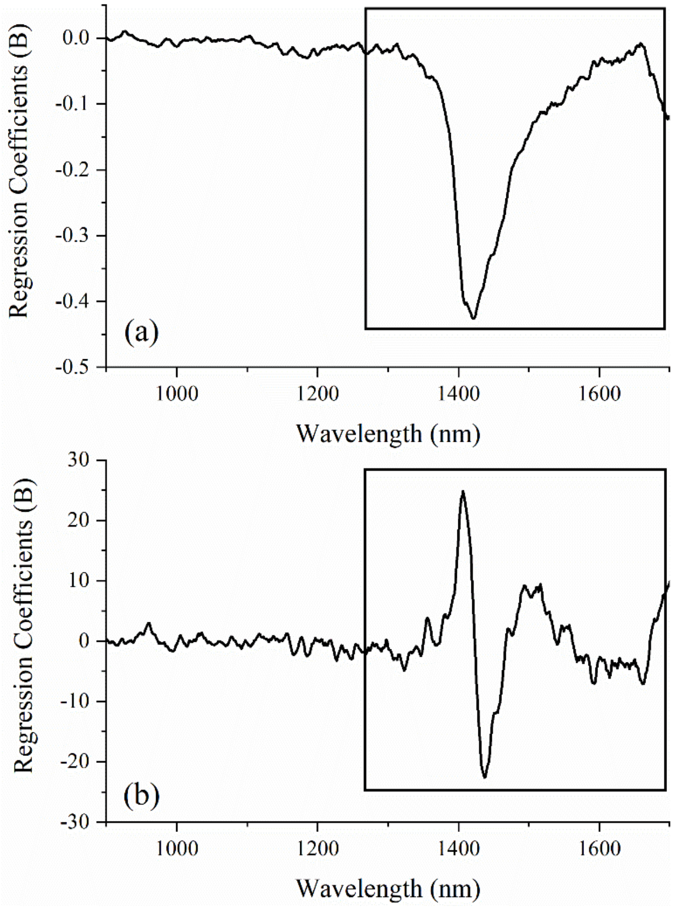

Figure S1. Regression coefficients for HNO_3 (**a**) concentration factor-4 and (**b**) temperature factor-5. The wavelengths with the most importance are outlined.

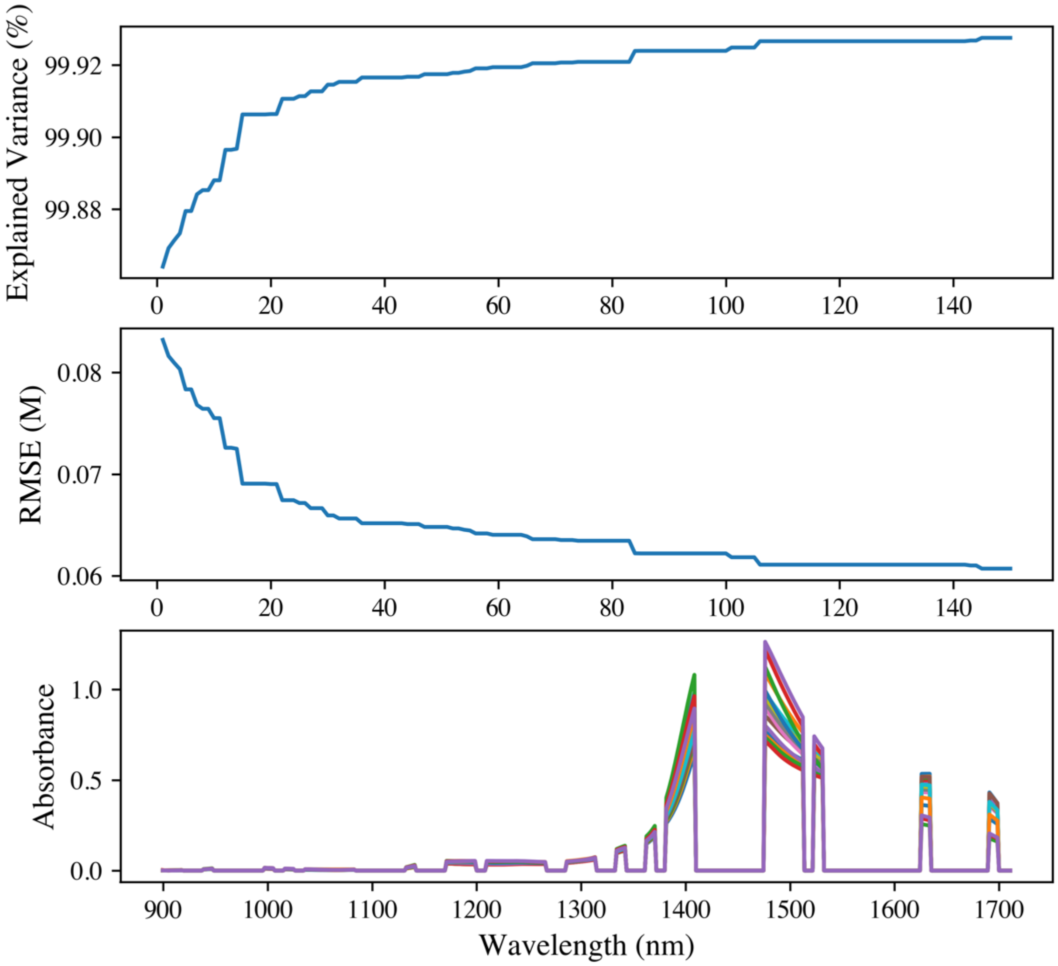

Figure S2. GA results for the HNO_3 model. The top two plots show the change in explained variance and RMSE, respectively, of the best filter in each generation of the GA. The bottom plot shows the spectral regions selected by GA for acid after preprocessing (smoothing).

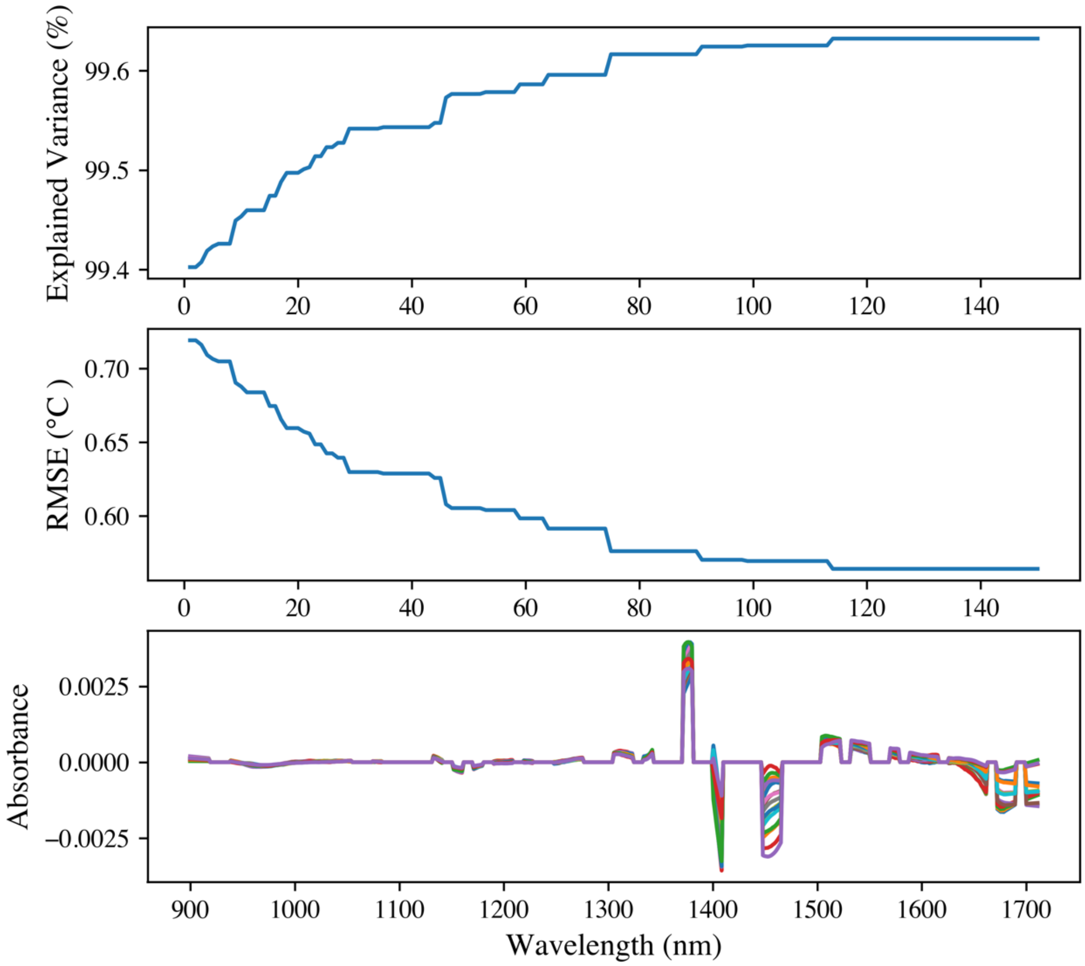

Figure S3. GA results for the temperature model. The top two plots show the change in explained variance and RMSE, respectively, of the best filter in each generation of the GA. The bottom plot shows the spectral regions selected by GA for temperature after preprocessing (scatter correction and smoothing/derivative).
